# The Compliance of Doctors with Viral Hepatitis B Screening and Antiviral Prophylaxis in Cancer Patients Receiving Cytotoxic Chemotherapy Using a Hospital-Based Screening Reminder System

**DOI:** 10.1371/journal.pone.0116978

**Published:** 2015-02-06

**Authors:** Wei-Chih Sun, Ping-I Hsu, Hsien-Chung Yu, Kung-Hung Lin, Feng-Woei Tsay, Huay-Min Wang, Tzung-Jiun Tsai, Wen-Chi Chen, Kwok-Hung Lai, Jin-Shiung Cheng

**Affiliations:** 1 Division of Gastroenterology, Department of Medicine, Kaohsiung Veterans General Hospital, Kaohsiung, Taiwan; 2 National Yang-Ming University, Taipei, Taiwan; 3 Department of Nursing, Meiho Institute of Technology, Ping-Tung, Taiwan; Kaohsiung Medical University Hospital, Kaohsiung Medical University, TAIWAN

## Abstract

**Background and Aim:**

Screenings for hepatitis B surface antigen (HBsAg) and antiviral prophylaxis are recommended for HBsAg-positive patients before the start of cytotoxic chemotherapy; however, compliance with these recommendations varies among doctors. We investigated the compliance of doctors with these recommendations using a reminder system and assessed the outcomes of HBsAg-positive patients receiving cytotoxic chemotherapy.

**Methods:**

Using a computer-assisted reminder system, doctors were alerted of both HBsAg screening and antiviral prophylaxis prior to prescribing chemotherapy. The compliance between different doctors and outcomes of patients were investigated during the period of execution of this system. The rates of compliance with both recommendations were compared among various cancer types.

**Results:**

A total of 1053 patients were enrolled, of which only 88 had previous data pertaining to HBsAg status. Using this reminder system, an overall screening rate of 85.5% (825/965) was achieved and did not significantly differ according to cancer type. However, the overall antiviral prophylactic rate was only 45.5% (61/134). The rates of antiviral prophylaxis were lower for doctors treating lung, breast and colorectal cancers than for those treating hematological malignancies (all p<0.05). Consequently, the rate of HBV reactivation was lower in patients who received antiviral prophylaxis than in those who did not (1.6% vs. 15.1%; p<0.01). Multivariate analysis revealed that male gender and antiviral prophylaxis were both related to reactivation of hepatitis B (p<0.05).

**Conclusions:**

By using this reminder system, the overall screening rate for HBsAg was satisfactory, whereas the antiviral prophylaxis was inadequate in patients with solid tumors due to the varying compliance of the attending doctors. Further strategies to improve both screening and prophylaxis are needed to minimize HBV-related events during cytotoxic chemotherapy.

## Introduction

Reactivation of hepatitis B virus (HBV) is a well-recognized, potentially lethal complication that occurs in chronic hepatitis B patients undergoing cytotoxic chemotherapy for malignant diseases. The initial reports of HBV reactivation involved patients receiving treatment for hematologic malignancies [1–3]. High rates of HBV reactivation (from 24–88%) have been recently recognized in hepatitis B surface antigen (HBsAg)-positive patients undergoing hematopoietic stem-cell transplantation and rituximab plus steroid combination chemotherapy for lymphoma [4–8]. Although the risk of HBV reactivation and its clinical significance in relation to chemotherapy in patients with solid tumors are unclear, their reactivation rate is thought to be lower than that associated with hematological malignancies. Patients with solid tumors typically have normal immune function and receive chemotherapy that is less immunosuppressive [9]. However, the HBV reactivation rates and related mortality rates have been reported to be 7－68% and 4－41%, respectively, in HBsAg-positive patients receiving cytotoxic chemotherapy [10–13].

The potential clinical consequences of HBV reactivation are hepatic failure or liver damage from reactivation of hepatitis and interruption of cancer treatment due to hepatic reserve status, the immunosuppressive capacity of chemotherapy, and several viral factors, such as baseline HBV DNA levels [4–8, 12–15]. Fortunately, antiviral prophylaxis with the oral nucleos(t)ide analog (NA) has been shown to be effective in reducing the rate of HBV reactivation [16–20]. A systematic review of 10 prospective trials has demonstrated a four-to-sevenfold decrease in the rate of HBV reactivation with lamivudine prophylaxis compared to controls [21]. In contrast, deferred lamivudine therapy in patients with reactivation of hepatitis has been associated with a mortality rate as high as 67% [22]. Therefore, some international guidelines recommend screening for HBsAg with or without the anti-hepatitis B core antibody (Anti-HBc) in all cancer patients prior to initiation of chemotherapy and prophylactic or of pre-emptive antiviral therapy with NAs in HBsAg-positive patients undergoing cytotoxic chemotherapy [23–25].

Effective prophylaxis is available but depends on HBV screening before chemotherapy. However, it has been reported that the screening rates for HBV infection before chemotherapy are as low as 14% to 34% [26–30]. Although routine screening for HBV before chemotherapy has been shown to be cost-effective [31], the compliance varies regarding doctors in charge of chemotherapy who can provide such screening. Indeed, some patients infected with HBV experience chemotherapy-related HBV reactivation and death because they did not receive HBV screening or prophylactic antiviral therapy before chemotherapy. Currently, educational intervention was the only one method and reported to result in only a modest improvement [28]. Moreover, the optimal means of improving the compliance of doctors with both recommendations remains unclear. To date, no studies were found in the relevant medical literature regarding techniques to improve oncologists’ compliance with HBV screening and/or antiviral prophylaxis.

HBV infection is endemic and is associated with carrier rates of approximately 17–20% in Taiwanese individuals [32]. The National Health Insurance of this country has subsidized prophylactic antiviral therapy for HBsAg—positive patients receiving chemotherapy since 2009. Universal screening for HBV infection prior to chemotherapy is important because many patients are unaware of their HBV status. Therefore, we established a computer—assisted, novel system for reminding clinicians in charge of chemotherapy to screen for HBsAg and to perform antiviral prophylaxis in HBsAg-positive patients prior to initiation of chemotherapy. Using this strategy, we aimed to increase the rates of both HBV screening and antiviral prophylaxis. Consequently, we designed this retrospective study to investigate the compliance of doctors with both recommendations using this reminder system. Rates of HBV reactivation as well as outcomes of HBsAg-positive patients undergoing chemotherapy and factors related to HBV reactivation were also investigated.

## Materials and Methods

### Study design and study population

**Fig 1:**
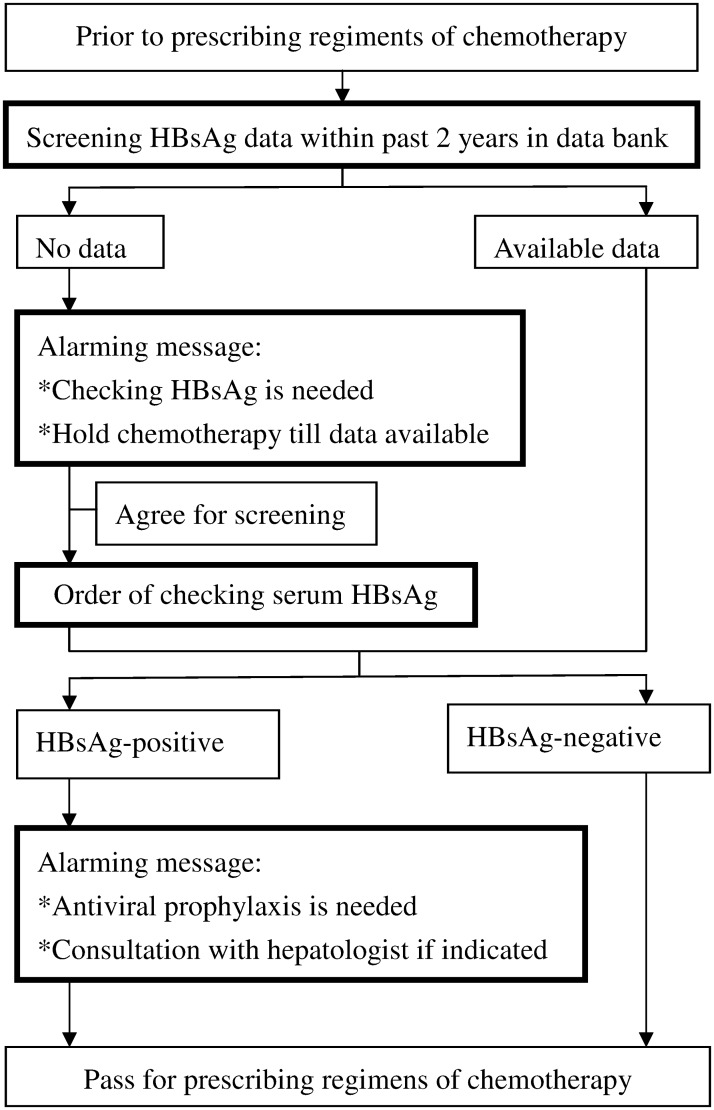
Flow chart of the strategy of computer-assisted system for reminding the doctors in charge before prescribing chemotherapy.

A retrospective review was conducted for all cancer patients undergoing cytotoxic chemotherapy at the Kaohsiung Veterans General Hospital from September 2011 to June 2012. We excluded those patients receiving targeted or hormonal therapy alone (for which without immunosuppressive effects) or localized (intra-peritoneal, intra-pleural, intra-bladder, etc.) chemotherapy. Patients with hepatitis C infection were not excluded but without detail analysis in this study. During this period, we set up a computer—assisted system for sending reminders to the doctor in charge of chemotherapy (Fig. 1). First, the computer performed an automatic screen of the laboratory database for HBsAg results obtained within past 2 years from patients preparing for chemotherapy at our hospital. For patients without previous HBsAg data, alerts were sent to remind the doctor in charge of chemotherapy to screen for HBsAg and to delay chemotherapy until the results were received. If the doctor selected the option to agree for screening, an order to test the serum HBsAg level was automatically sent. In addition, if HBsAg—positivity was noted in either the past data or in the screening results, additional alerts were sent to the doctor in charge, suggesting antiviral therapy. Antiviral therapy with NA was initiated following consultation of the doctor with hepatologists. The compliance of doctors with regard to screening for HBsAg in patients with unknown HBsAg status and prescribing antiviral prophylaxis to HBsAg-positive patients prior to chemotherapy were investigated.

### Ethics statement

This study was approved by the Ethics Committee and the Institutional Review Board of the Kaohsiung Veterans General Hospital (VGHKS13-CT6–12). This was a retrospective study that did not involve patient intervention or the need for obtaining clinical specimens, and all of the data were analyzed anonymously. Therefore, informed consent was waived. The waiving of informed consent was approved by the Kaohsiung Veterans General Hospital Institutional Review Board.

### Data collection and follow up

The basic characteristics, cancer type, chemotherapy regimen, status of hepatitis B reactivation and antiviral prophylaxis for all patients undergoing chemotherapy were collected. For those patients receiving antiviral therapy, a liver function test and assessments of hepatitis e antigen (HBeAg) and HBV DNA levels were conducted at baseline, every 3 months during treatment and after discontinuation of the drugs. In general, antiviral therapy was discontinued if cessation of chemotherapy occurred over 6 months prior. The selection of NAs for antiviral therapy, including lamivudine, telbivudine, entecavir or tenofovir, was performed according to the hepatologist in charge. Patients receiving antiviral therapy were followed in the clinic of the hepatologist.

### Definition of HBV reactivation

HBV reactivation was defined by a combination of two criteria as follows: (1) virological criteria, including an increase in HBV DNA of more than 1 log IU/ml compared to nadir, a reappearance of HBV DNA and/or HBeAg in the serum or an absolute HBV DNA level of over 20000 IU/ml if nadir data were not available; (2) clinical criteria, including an increase in alanine transaminase (ALT) to over 3 times the upper limit of normal in patients with a normal baseline ALT level or an increase of more than 100 U/L compared to nadir in patients with abnormal baseline ALT or development of hepatic decompensation (total bilirubin > 2 mg/dl and/or prothrombin time prolongation of > 3 seconds). For patients without antiviral prophylaxis experiencing HBV reactivation, salvage antiviral therapy with NA was initiated as soon as possible. For patients with antiviral prophylaxis who developed HBV reactivation, the genotypic resistance of HBV to NAs and drug compliance were assessed. Salvage therapy was accordingly administered if genotypic resistance to NAs was detected.

### Measurements of HBV markers and DNA

HBV DNA levels were measured using an Abbott Real Time HBV assay (Abbott Molecular Inc., Des Plaines, IL, USA) with a lower detection limit of 10 IU/ml. Genotypic resistance of HBV was determined by direct DNA sequencing (SeqHepB; Abbott Diagnostics, Lake Forest, IL, USA). Serum HBsAg, HBeAg, and anti-HBe antibodies were measured using radioimmunoassay kits (Ausria II-125; Abbott Laboratories, North Chicago, IL, USA).

### Statistical Analysis

All statistical analyses were performed using Statistical Program for Social Sciences (SPSS 12.0 for Windows; SPSS Inc., Chicago, IL, USA). Pearson chi-squared analysis or Fisher’s exact test was used for comparison of categorical variables, while continuous variables were compared using Student’s t-test or the Mann-Whitney U test as appropriate. Logistic regression models were used to estimate the factors related to reactivation of hepatitis B. Variables with marginal statistical significance (*P* < 0.1) in the univariate analysis were subjected to multivariate analysis. A two-tailed *p* value of < 0.05 was considered significant for all tests.

## Results

A total of 1053 cancer patients were enrolled. Their demographic data are summarized in Table 1. A total of 40 (3.8%) patients had liver cirrhosis related to HBV infection (n = 24), HCV infection (n = 13), alcohol (n = 2), and cryptogenic etiology (n = 1). Approximately 18.2% of the patients had abnormal baseline ALT levels. There were 2 hemato-oncologists, 4 hepatogastroenterologists, 9 pulmonologists, 1 infectious disease physician, 4 colorectal surgeons, 9 general surgeons, 3 thoracic surgeons, 8 gynecologists, 6 otolaryngologists, 2 radiation oncologists, 1 dermatologist, and 1 dentist. The majority (98.5%) of doctors in charge were not hepatologists. Approximately 91.6% of the patients had solid tumors other than HCC or hematological malignancy. The major types of cancer were breast (22.2%), lung (21.7%) and colorectal (19.8%) cancers. In addition, 91.8% of the patients received steroid- and rituximab-free regimens.

**Table 1:** Demographic data of cancer patients in overall (n = 1053) who receiving chemotherapy shown by baseline characteristics, departments of doctors, cancer types and regimens of chemotherapy.

Characteristics	Patients (n, %)
Age, years (mean ± standard deviation)	57.7 ± 13.2
Sex: Male / Female	479 (45.5) / 574 (54.5)
Cirrhosis	40 (3.8)
Liver metastasis	109 (10.4)
Baseline ALT levels (U/L)	
≦ 40 / 40–80 / 80–200 / > 200	861 (81.8) / 152 (14.4) / 37 (3.5) / 3 (0.3)
HBsAg status in previous 2 years: known / unknown	88 (8.4) / 965 (91.6)
Anti-HCV Ab status in previous 2 years: known / unknown	85 (8.1) / 968 (91.9)
Departments of doctors	
Medical department	291 (27.6)
Hemato—oncology (2 doctors)	107 (10.2)
Hepatogastroenterology (4 doctors)	16 (1.5)
Chest medicine (9 doctors)	166 (15.8)
Others (Infection) (1 doctor)	2 (0.2)
Surgical department	573 (54.4)
Colorectal surgery (4 doctors)	199 (18.9)
General surgery (9 doctors)	280 (26.6)
Thoracic surgery (3 doctors)	94 (8.9)
Gynecology (8 doctors)	88 (8.4)
Otolaryngology (6 doctors)	86 (8.2)
Others (Dentalogy, Dermatology, Radioncology) (4 doctors)	15 (1.4)
Cancer types	
Hematological cancer	50 (4.7)
Hepatocellular carcinoma	39 (3.7)
Solid tumor other than hepatocellular carcinoma	964 (91.6)
Lung cancer	228 (21.7)
Colorectal cancer	209 (19.8)
Breast cancer	234 (22.2)
Gynecological cancer	84 (8.0)
Head and Neck cancer	100 (9.5)
Urological cancer	17 (1.6)
Gastroenterological cancer except for colorectal cancer	48 (4.6)
Biliary and Pancreatic cancer	20 (1.9)
Others	24 (2.3)
Regimens of chemotherapy	
Steroid—contained regimen	50 (4.7)
Combined Rituximab and steroid regimen	36 (3.4)
Others	967 (91.8)

ALT, alanine transaminase; HBsAg, hepatitis B surface antigen; HCV Ab, hepatitis C virus antibody. ALT, alanine transaminase; HBsAg, hepatitis B surface antigen; HCV Ab, hepatitis C virus antibody.

**Fig 2:**
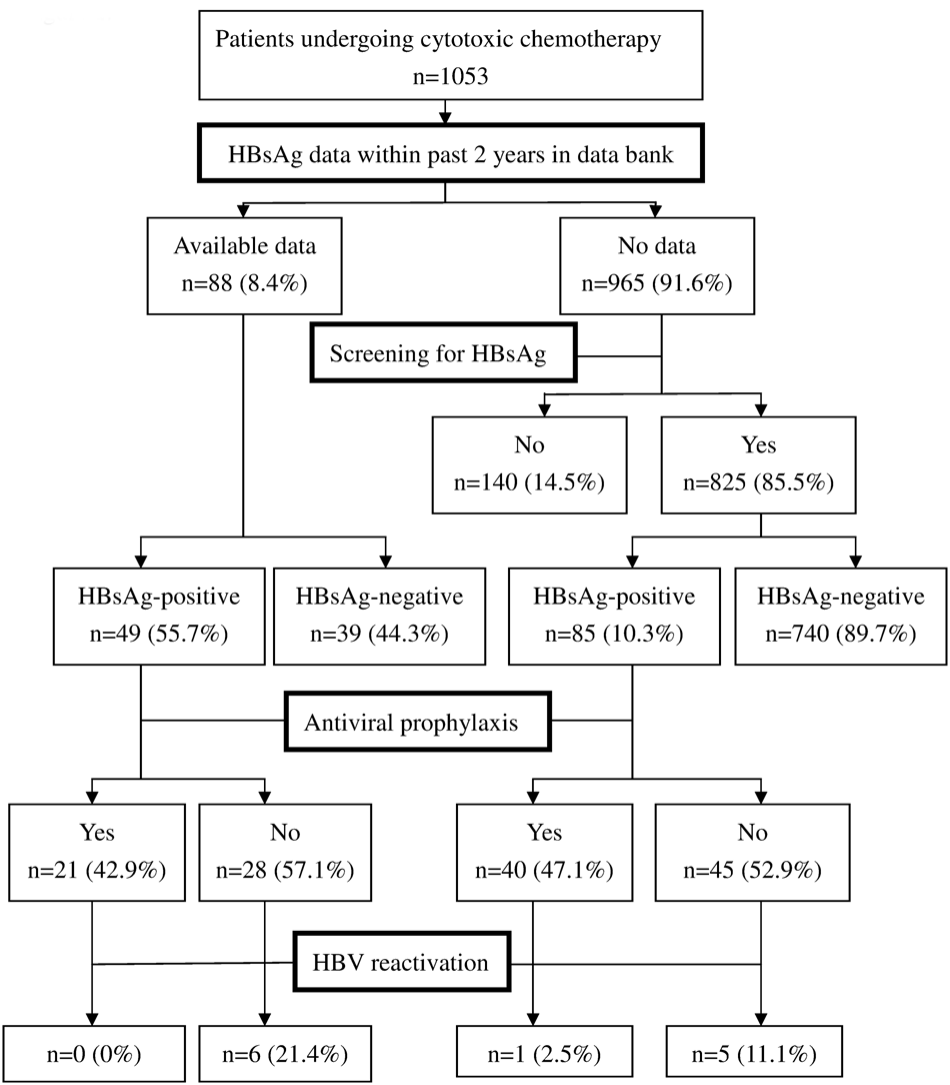
Flow chart of patients with or without screening for hepatitis B surface antigen (HBsAg), antiviral prophylaxis and hepatitis B virus reactivation in overall (n = 1053) by way of the computer－assisted reminding system.

**Fig 3:**
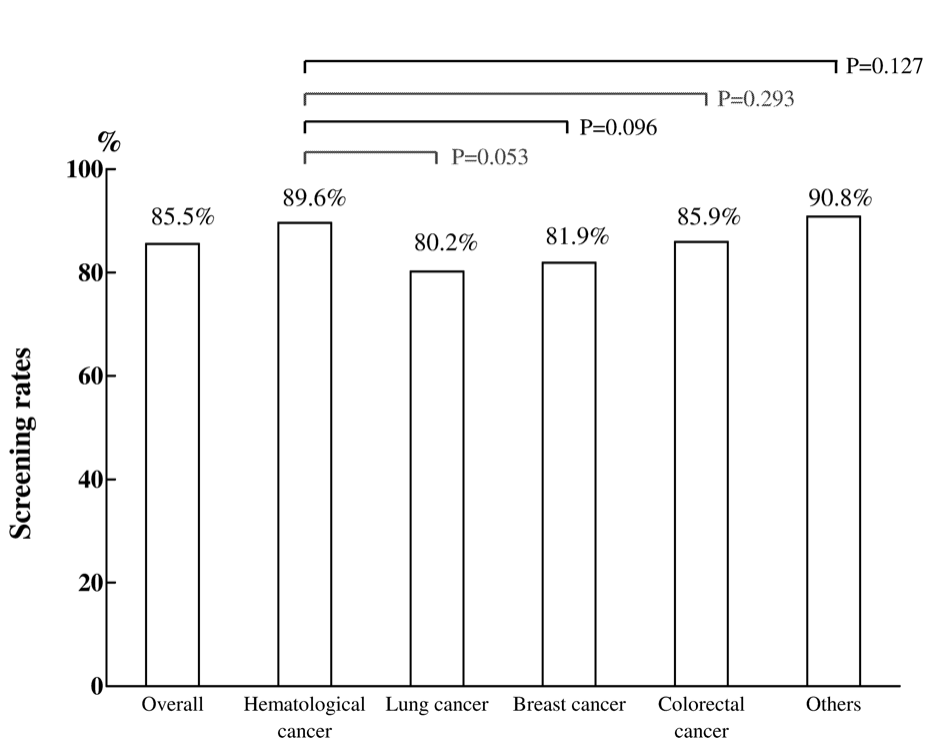
Screening rates for hepatitis B surface antigen in patients without previous data (n = 965) before chemotherapy in different cancer types. The screening rates were similar in patients with different cancer types through this assisted system.

As shown in Fig. 2, 88 (8.4%) patients had been tested for HBsAg within the past 2 years prior to chemotherapy. Among the 965 patients without previous data, the screening rate for HBsAg was 85.5% (825 of 965), which was higher than the previous screening rate of 26.8% in 2009, which was measured before the reminder system was initiated at our hospital [34]. As shown in Fig. 3, the reminder system led to screening rates of over 80% for the majority of the doctors assessed. Moreover, the screening rates were similar among cancer types (all p > 0.05).

**Fig 4:**
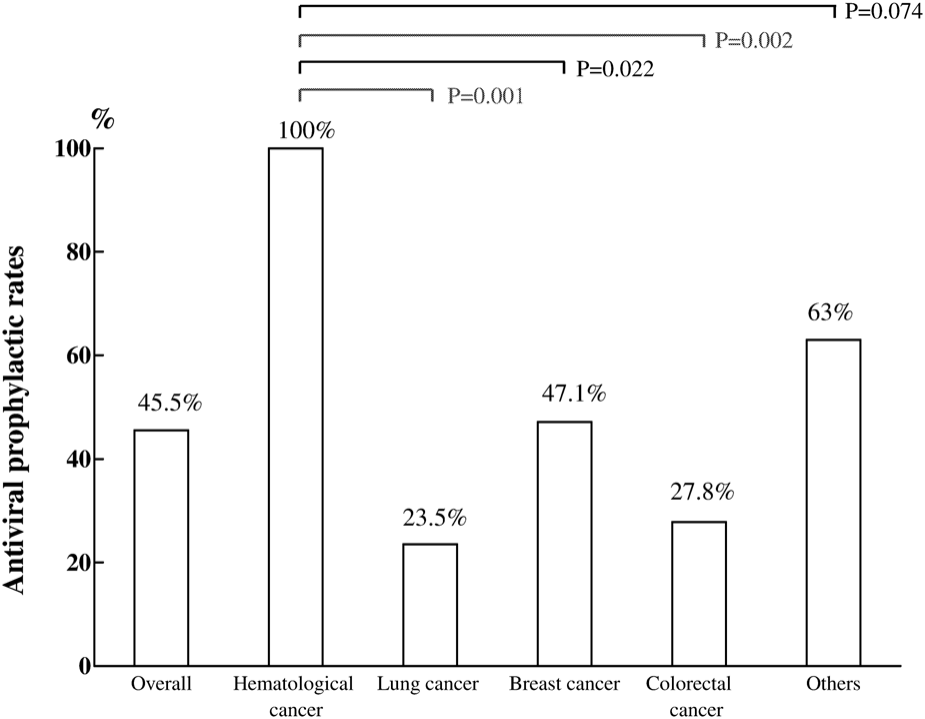
Antiviral prophylactic rates in patients with positive of hepatitis B surface antigen (n = 134) undergoing chemotherapy in different cancer types. The rates of antiviral prophylaxis in doctors treating lung, breast and colorectal cancers were significantly lower in doctors treating hematological cancers (all p < 0.05).

A total of 86.7% (913 of 1053) of the patients had HBsAg data that were reported prior to initiation of chemotherapy, and 14.6% (134 of 913) of these patients tested positive for HBsAg (Fig. 2). However, as shown in Fig. 4, the overall rate of antiviral prophylaxis was only 45.5% (61 of 134). Nevertheless, the doctors treating hematologic malignancy achieved 100% antiviral prophylaxis. In contrast, rates of antiviral prophylaxis were lower for the doctors treating lung (23.5%), colorectal (27.8%) and breast cancers (47.1%) compared with those treating hematological malignancy (all p < 0.05). For the 61 HBsAg-positive patients who received antiviral prophylaxis, 24 (39.3%) were administered lamivudine, 5 (8.2%) received telbivudine, 15 (24.6%) received entecavir, and 17 (27.9%) were administered tenofovir.

Among the 134 HBsAg-positive patients, 12 (including 5 with lung cancer, 4 with colorectal cancer, 1 with HCC, 1 with ovarian cancer, and 1 with gastric cancer) developed HBV reactivation. The rates of HBV reactivation were higher in the patients who did not receive antiviral prophylaxis compared with those who did receive this treatment (15.1% vs. 1.6%; p < 0.01) (Table 2). Multivariate analysis revealed that male gender (hazard ratio: 9.86, 95% CI: 1.18–82.16, p = 0.03) and antiviral prophylaxis (hazard ratio: 0.10, 95% CI: 0.01–0.82, p = 0.03) were two factors related to HBV reactivation (Table 3). However, when we restricted this analysis to the patients without antiviral prophylaxis (n = 73), none of the clinical factors were related to HBV reactivation by univariate analysis (not shown). Nevertheless, only some of patients in hematological department received forced chemotherapy including agents with strong immunosuppressive effects such as high-dose steroid as well as second- or third-line agents; besides, all of these patients had antiviral prophylaxis and none of them had HBV reactivation. Therefore, forced chemotherapy was not included in analysis of risk factors for HBV reactivation. For the 140 patients who had not been tested for HBsAg, 12 (8.6%) experienced an increase in their ALT level during chemotherapy. However, none of the ALT levels were sufficiently elevated to meet the criteria for reactivation of hepatitis in the present study. Consequently, the cause of this increase in ALT was not confirmed to be hepatitis B for these patients.

**Table 2:** Clinical data of 11 patients with hepatitis B reactivation without antiviral prophylaxis before chemotherapy.

Case Number	Cancer type	Department of doctor	BaselineALT (U/L)	Peak ALT (U/L)	Hepatic decompensation	HBV DNA on reactivation (IU/ml)	Courses of C/T before reactivation	Time from last C/T to reactivation (days)	Salvage treatment of reactivation	Outcome
1	Colon cancer	Colorectal surgery	18	118	No	602000	10	8	ETV 0.5mg	Recovery
2	Lung cancer	Chest medicine	78	955	No	3950000	1	25	ETV 0.5mg	Recovery
3	Lung cancer	Thoracic surgery	36	303	Yes	21972	5	56	ETV 0.5mg	Recovery
4	Colon cancer	Colorectal surgery	31	121	No	26000	7	10	ETV 0.5mg	Recovery
5	HCC	General surgery	44	366	No	11853	2	39	LdT 600mg	Recovery
6	Gastric cancer	General surgery	29	880	Yes	4326631	5	30	LdT 600mg	Expired
7	Lung cancer	Thoracic surgery	30	217	No	10372	5	7	LAM 100mg	Recovery
8	Lung cancer	Thoracic surgery	87	372	Yes	61000	3	21	TDF 300mg	Recovery
9	Rectal cancer	Colorectal surgery	21	494	No	> 10 ^10^	4	17	TDF 300mg	Recovery
10	Lung cancer	Chest medicine	22	115	No	10126	2	8	LAM 100mg	Recovery
11	Rectal cancer	Colorectal surgery	18	468	Yes	NA	10	30	No	Expired

ALT, alanine aminotransferase; HCC, hepatocellular carcinoma; NA, not available; C/T, chemotherapy; ETV, entecavir; LdT, telbivudine; LAM, lamivudine; TDF, tenofovir.

All patients were hepatitis B e antigen-negative.

Case 5, Case 7, Case 10 reported themselves to be inactive HBV carriers in other medical institutions.

Case 11 died within 2 days after admission without virological data and antiviral treatment.

**Table 3:** The rates of reactivation of hepatitis B and related events in patients (n = 134) with and without antiviral prophylaxis before prescribing chemotherapy.

	With prophylaxis	Without prophylaxis	P value
	(n = 61)	(n = 73)	
Reactivation of hepatitis B	1 (1.6%)	11 (15.1%)	< 0.01
Hepatic decompensation	0 (0%)	4 (5.5%)	0.13
Hepatitis B related delay of chemotherapy	1 (1.6%)	11 (15.1%)	< 0.01
Hepatic failure and mortality	0 (0%)	2 (2.7%)	0.50

Finally, all of the patients (n = 12) with HBV reactivation experienced a delay in their scheduled chemotherapy. In addition, 4 of them (33%) progressed to hepatic decompensation, and 2 (17%) died of hepatic failure within 2 months. The clinical data of 11 patients without antiviral prophylaxis and with HBV reactivation are shown in Table 4. Active treatment with a nucleos(t)ide analog for HBV reactivation was performed for all patients except case 11, who developed fulminant hepatic failure with encephalopathy and died within 2 days after admission; thus, this patient was were able to receive a further complete evaluation or antiviral treatment. The rates of hepatic decompensation and mortality did not achieve statistical significance in the patients with and without antiviral prophylaxis (both p > 0.05) (Table 2). Nevertheless, none of the patients with antiviral prophylaxis experienced fulminant hepatic failure with encephalopathy or death. Only one patient experienced HBV reactivation in the prophylactic group. She received antiviral prophylaxis by tenofovir. However, HBV reactivation developed at week 24, which resulted from poor drug compliance rather than genotypic resistance after series check-up. Consequently, normalization of liver function and negativity of HBV DNA were achieved by continuous tenofovir therapy over additional 3 months.

**Table 4:** Factors related to reactivation of hepatitis B in patients with positive of hepatitis B surface antigen (n = 134) undergoing cytotoxic chemotherapy.

Risk factor	Univariate	p value	Multivariate	p value
	HR (95% CI)		HR (95%CI)	
Age (years)	1.01 (0.96–1.06)	0.66		
Sex: male	7.90 (0.99–63.15)	0.05	9.86 (1.18–82.16)	0.03
Cancer types	0.87 (0.56–1.33)	0.52		
Cirrhosis	0.41 (0.05–3.37)	0.41		
Anti-HCV antibody: positive	2.13 (0.23–19.87)	0.51		
Liver metastasis	1.29 (0.57–2.89)	0.55		
Baseline ALT > 40 IU/mL	2.13 (0.65–7.02)	0.22		
Baseline HBV DNA > 2000 IU/mL	2.12 (0.58–7.78)	0.26		
HBeAg: positive	0.00	0.99		
Antiviral prophylaxis	0.09 (0.01–0.75)	0.03	0.10 (0.01–0.82)	0.03
Chemotherapy agents				
Alkylating agents	0.89 (0.23–3.53)	0.87		
Anti-metabolites	0.49 (0.14–1.76)	0.28		
Antibiotics	0.32 (0.07–1.52)	0.15		
Anti-microtubule agents	0.74 (0.15–3.58)	0.71		
Topoisomerase inhibitors	4.41 (1.26–15.50)	0.02	3.74 (0.92–15.14)	0.07
Target therapy except for rituximab	1.33 (0.27–6.61)	0.73		

ALT, alanine transaminase; HBeAg, hepatitis B e-antigen; HR, hazard ratio.

## Discussion

HBV reactivation is a potentially fatal complication of chemotherapy that can be largely prevented with antiviral prophylaxis. Effective prophylaxis is available but depends on the detection of prior HBV infection. However, although they are knowledgeable of the guideline recommendations, most doctors do not take the initiative to arrange for HBV infection testing or to carry out prophylactic antiviral therapy for HBsAg-positive patients prior to chemotherapy [33]. Educational intervention has failed to increase the rates of HBV infection screening and antiviral prophylaxis due to unawareness of current guidelines and a perception that HBV reactivation does not occur with solid tumors [28]. Nevertheless, this study has provided a means of improving the HBsAg screening rate before chemotherapy in clinical practice. Our results clearly demonstrated that we were able to increase the screening rate using a novel computer-assisted reminder system at our hospital. Through this strategy, screening prior to initiation of chemotherapy was achieved in 85.5% cases, which is higher than our previous screening rate of 26.8% that was measured in 2009 (when the reminder system had not yet been initiated) [34]. This study has achieved the highest screening rate reported in the literature world-wide. However, although the screening rate has been improved from the convenience of this reminder system for HBV screening, ideal 100% screening rate is not achieved, which may result from the poor compliance of doctors. Further strategies, such as setting up a mandatory system for HBV screening, are considered in the feature.

In addition, our results clearly showed that antiviral prophylaxis with NAs was the most effective means of reducing HBV-related events in patients undergoing cytotoxic chemotherapy. However, antiviral prophylactic rates were inadequate in patients with solid tumors, particularly in those with lung, colorectal and breast cancers. The doctors treating solid tumors showed a low compliance with regard to this issue in the present study. Although high screening rates (over 80%) were achieved for HBsAg, antiviral prophylactic rates were approximately 23.5% to 47.1% for the three major cancers reported at our hospital. Consequently, approximately 15.1% of the patients without antiviral prophylaxis experienced HBV reactivation, which is a theoretically preventable event. Moreover, 4 patients developed severe hepatic decompensation, and 2 died of hepatic failure because of HBV reactivation. Unfortunately, we could not determine the factors underlying this HBV reactivation in the patients without antiviral prophylaxis in this study. Thus, there was no clear characteristic, such as cancer type, chemotherapy regimen or other clinical indication, which could have facilitated the safe administration of pre-emptive antiviral therapy to these patients. Although neither severe event achieved statistical significance in the patients with versus those without antiviral prophylaxis, administration of antiviral prophylaxis was still the safest means of minimizing HBV-related hepatic decompensation and mortality in clinical practice.

At the same time, our computer-assisted system did not improve the rate of antiviral prophylaxis before chemotherapy for the doctors treating solid tumors. This discrepancy between the high screening rates and inadequate prophylactic rates may have resulted from the incomplete flow path of this system as well as the low compliance of the doctors in charge. Indeed, through the automatic delivery of the order for HBsAg serum screening through this assisted system, the screening rates were improved. In contrast, the antiviral prophylactic rates did not improve because no further control or setting was put in place to address the issue of ignored messages. In fact, this reminder system requires that doctors spend more time consulting with hepatologists for prescribing antiviral agents compared with time spent if the reminders are ignored. Although further strategies for optimizing the flow path for ease of use are needed, setting a therapeutic control system is more considered to enforce consultation of hepatologists for antiviral prophylaxis and to block chemotherapy until antiviral agents available. In addition, a subsequent study to assess why doctors treating patients with solid tumors are unlikely to be compliant may be also needed.

Nearly all of the hemato-oncologists screened for HBsAg and performed prophylactic antiviral therapy in this study, which was likely because they had more previous experience with chemotherapy-related HBV reactivation in daily practice [35, 36]. Because there are substantially more reports in the literature describing HBV reactivation in patients with hematologic malignancies compared to those with solid tumors [8, 17, 37], the screening and antiviral prophylactic rates were higher for the doctors treating hematologic cancers than for those treating solid tumors in this study. Moreover, according to the present study, most doctors treating non-hematologic malignancy had little experience of chemotherapy-induced HBV reactivation in the past. Hence, lack of popularization and ignorance of importance about this issue are the major reasons why the prophylaxis rate is low. Nevertheless, more and more studies have shown chemotherapy-induced HBV reactivation in patients with solid tumors [38–40]. Changes in the attitudes and behaviors of doctors treating solid tumors with regard to HBV screening and antiviral prophylaxis can be foreseen.

In this study, all of the patients in the non-prophylactic group who experienced HBV reactivation were male. This discrepancy may be due to the predominance of male patients with lung and colorectal cancers, who comprised 75% of the patients with HBV reactivation. In contrast, none of the patients with breast or gynecological cancers exhibited HBV reactivation, including those without antiviral prophylaxis. According to previous studies, rates of HBV reactivation vary drastically (from 14% to 68%) because of the different definitions of HBV reactivation that exist [11, 41–44]. An age of over 55 years and adverse liver fibrosis or cirrhosis are considered to be independent risk factors for HBV reactivation in patients with breast cancer [44], but they were not shown to be significant in this study. The discrepancy may be because our patients were younger (median age 47 years) and did not have cirrhosis or advanced fibrosis. In addition, the definition of HBV reactivation in this study, which included both virological and clinical criteria, was stricter than that in other studies. However, the role of gender in HBV reactivation requires further study.

There are some limitations of this study. First, due to its retrospective nature, we did not have data pertaining to HBV genotypes, HBV mutations or quantitative HBsAg levels for further analysis. The impacts of viral factors on HBV reactivation were not well studied. Second, none of the patients with a past history of resolved HBV (positive for anti-HBc but negative for HBsAg with or without anti-HBs) were assessed for their risk of *de novo* HBV flare during cytotoxic chemotherapy, but this study did not deal with this issue Nevertheless, in our hospital, the doctors treating hematological malignancy typically perform antiviral prophylactic therapy for nearly all of these types of patients receiving steroid and rituximab combination chemotherapy because of the high risk of *de novo* HBV reactivation [45, 46]. In addition, in patients with solid tumors, *de novo* HBV reactivation is extremely rare during cytotoxic chemotherapy [8]. Indeed, due to a potential of HBV reactivation among cancer patients with occult HBV infection during chemotherapy, further strategies for prevention of *de novo* HBV reactivation in these patients are needed especially in high risk patients according to guideline suggestion [23]. Finally, the doctors’ level of expertise in general (e.g. number of years in practice) and/or knowledge level and exposure to HBV in oncology patients in specific, might play a role in their compliance. However, it was hard to address the area in this study. These issues can be improved in future studies.

In conclusion, a satisfactory overall HBsAg screening rate was observed using this reminder system. However, antiviral prophylaxis was inadequate in patients with solid tumors because the compliance of the doctors in charge with carrying out this treatment was quite variable. Antiviral prophylaxis is still the safest means of minimizing HBV-related events that occur during chemotherapy in clinical practice. For further improvements, the use of mandatory barriers for screening and antiviral prophylaxis may be the most optimal means of improving the compliance of doctors in charge and preventing HBV-related events during chemotherapy.
